# Putative anticancer potential of novel 4-thiazolidinone derivatives: cytotoxicity toward rat C6 glioma *in vitro* and correlation of general toxicity with the balance of free radical oxidation in rats

**DOI:** 10.3325/cmj.2016.57.151

**Published:** 2016-04

**Authors:** Lesya I. Коbylinska, Nataliya M. Boiko, Rostyslav R. Panchuk, Iryna I. Grytsyna, Olga Yu. Klyuchivska, Liliya P. Biletska, Roman B. Lesyk, Borys S. Zіmenkovsky, Rostyslav S. Stoika

**Affiliations:** 1 Department of Biochemistry, Danylo Halytsky Lviv National Medical University, Lviv, Ukraine; 2 Department of Regulation of Cell Proliferation and Apoptosis, Institute of Cell Biology, NAS of Ukraine, Lviv, Ukraine; 3 Department of Pharmaceutical Chemistry, Danylo Halytsky Lviv National Medical University, Lviv, Ukraine

## Abstract

**Aim:**

To evaluate the cytotoxic action of 4-thiazolidinone derivatives (ID 3288, ID 3882, and ID 3833) toward rat glioma C6 cells and to compare the effects of these compounds and doxorubicin on the balance of free radical oxidation (FRO) and antioxidant activity (AOA) in the serum of rats.

**Methods:**

Glioma cells were treated with ID 3882, ID 3288, ID 3833, and doxorubicin, and their cytotoxicity was studied using MTT (3-(4,5-dimethylthiazol-2-yl)-2,5-diphenyltetrazolium bromide) assay and Trypan blue exclusion test, light and fluorescent microscopy, and flow cytometric study of cell cycling and apoptosis, including measuring of Annexin V-positive cells. The contents of superoxide radical, hydrogen peroxide, hydroxyl radical, malonic dialdehyde, and hydrogen sulfide were measured in the serum of rats. Enzymatic activity of superoxide dismutase (SOD), catalase (Cat), and glutathione peroxydase (GPO) was determined.

**Results:**

Among novel 4-thiazolidinone derivatives, ID 3288 was most toxic toward rat glioma C6 cells, even compared with doxorubicin. All applied derivatives were less active than doxorubicin in inducing reactive oxygen species-related indicators in the serum of rats. A similar effect was observed when enzymatic indicators of AOA processes were measured. While doxorubicin inhibited the activity of SOD, GPO, and Cat, the effects of 4-thiazolidinone derivatives were less prominent.

**Conclusion:**

Novel 4-thiazolidinone derivatives differ in their antineoplastic action toward rat glioma C6 cells, and ID 3288 possesses the highest activity compared to doxorubicin. Measurement of indicators of FRO and AOA in the serum of rats treated with these compounds showed their lower general toxicity compared with doxorubicin’s toxicity.

Chemotherapy is one of the most effective ways of treating cancer patients. Chemotherapeutic drugs suppress proliferation or irreversibly impair tumor cells via a direct interaction with the nucleic acids or enzymes that are responsible for their synthesis or functioning (1). Generally, these drugs impair rapidly proliferating cells, however they do not possess enough selectivity regarding their cell targets. Thus, their application in cancer treatment is accompanied by frequent non-addressed actions leading to numerous negative side effects in the organism (1-3). Due to these effects, they demonstrate toxicity toward different normal cells in tissues and organs, among which there are the bone marrow cells, mucous layer of the intestine, reproduction glands, and hair follicles. Although the list of clinically used anticancer drugs is rather long, a search for new drugs continues and, currently, many new drugs are at different phases of preclinical and clinical trials (4).

The anticancer potential of synthetic derivatives of heterocyclic 4-thiazolidinones was approved by the Development Therapeutics Program of screening new anticancer compounds at the National Сancer Institute (USA) (4). Our previous study of anticancer activity of the 4-thiazolidinones, including pyrazoline-substituted compounds, showed that pyrazoline-thiazolidinone-indoline conjugates were the most promising candidates for further pre-clinical study, and the compounds denoted as ID 3288, ID 3833, and ID 3882 were the most active among them (4,5). Their structure is shown in Figure 1, and their molar masses are 559.44 (ID 3288), 530.61 (ID 3882), and 609.51 g/mol (ID 3833). The main structural feature of these compounds is the presence of Br atom in the isatin fragment (5th position of ID 3288 and ID 3833) and substitution of the phenyl substituent (ID 3288) in the 3rd position of the pyrazoline cycle by the naphtyl fragment (ID 3833 and ID 3882) (4,5). These specific fragments might have a decisive influence on the cytotoxic action of the compared compounds. Therefore, the compounds ID 3288, ID 3833, and ID 3882 were selected for further in-depth *in vitro* and *in vivo* studies (4,6,7). They are structurally similar, belong to the patented group of the pyrazoline-thiazolidinone-isatins, and possess the antineoplastic activity toward cultured mammalian tumor cells. It should be stressed that they demonstrated lower general toxicity *in vivo* compared with the toxicity of doxorubicin (2,3,8).

**Figure 1:**
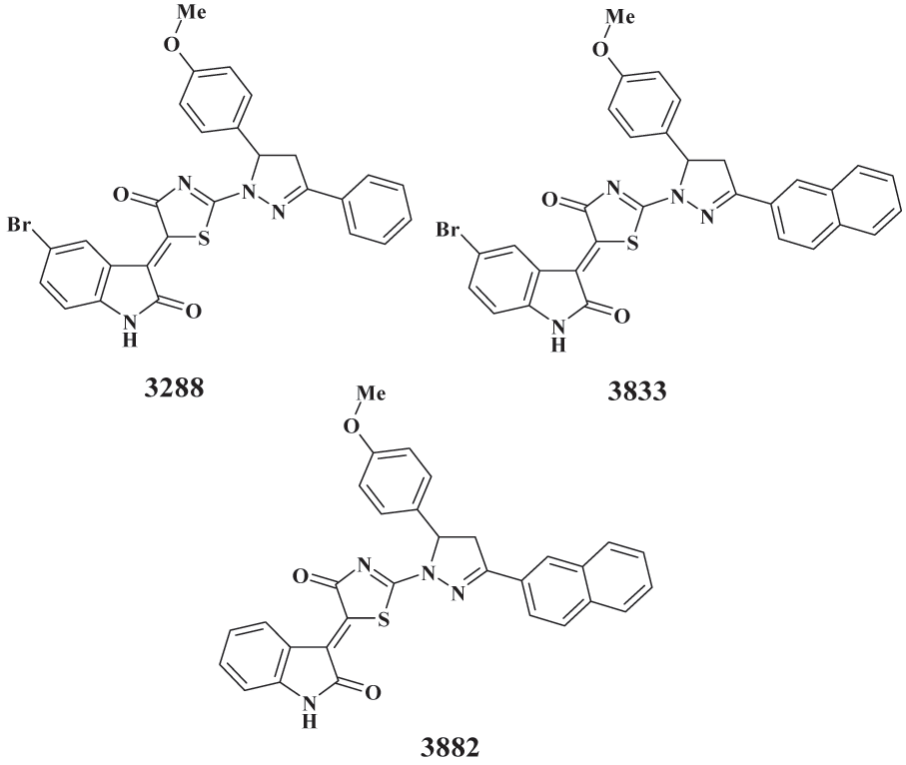
Structure of the studied 4-thiazolidinone derivatives – compounds ID 3882, ID 3288 and ID 3833.

The biochemical mechanisms responsible for a lower general toxicity of studied 4-thiazolidinones derivatives compared with doxorubicin have not yet been explained. Here we demonstrated that the compounds ID 3288, ID 3833, ID 3882 and doxorubicin differentially affected the balance of free radical oxidation (FRO) and antioxidant activity (AOA) in the target cells, which could be a reason of their different toxicity.

It is known that the action of many anticancer drugs is accompanied by an elevated production of reactive oxygen species (ROS), which are toxic for both normal and malignant cells (1,9,10). At the same time, malignant cells are characterized by the innate high level of ROS, which are considered to be the promoters of tumor progression (11,12). In order to neutralize the negative effects of ROS, tumor cells possess the mechanisms of antioxidant defense, and, thus, a balance of pro- and antioxidants exists in tumor (10-12). The redox adaptation through up-regulation of the anti-apoptotic and antioxidant molecules allows cancer cells to promote survival and develop resistance to anticancer drugs (10,12,13). The deterioration of such adaptation, for example by decreasing their antioxidant capacity by means of specific anticancer drugs, is a perspective way of enhancing the effectiveness of drug action (1,11).

Recently, we have found that the injection of compounds ID 3882, ID 3288, and ID 3833 to experimental rats is accompanied by a decrease in biochemical indicators of the negative side effects, such as cardio-, hepato-, and nephrotoxicity, although their magnitude was lower than in the case of doxorubicin (2,3,8).

The aim of present study was to determine the toxicity of novel 4-thiazolidinone derivatives (compounds ID 3288, ID 3882, and ID 3833) and doxorubicin (positive control) toward rat glioma C6 cells. Besides, the role of ROS measured in the serum of rats treated with the above mentioned compounds and doxorubicin was evaluated, and the enzymatic activity of superoxide dismutase (SOD), catalase (Cat), and glutathione peroxydase (GPO) was determined.

## Materials and methods

*Drugs.* The heterocyclic 4-thiazolidinones derivatives (compounds ID 3288, ID 3882 and ID 3833) were synthesized at the Department of Pharmaceutical, Organic and Bioorganic Chemistry of Danylo Halytsky Lviv National Medical University in 2012, as described (4,5). Before use, they were dissolved in the dimethyl sulfoxide (DMSO, Arterium, Lviv, Ukraine), and then additionally dissolved in distilled water before use. The final concentration of the DMSO in the medium of cultured cells was below 0.1%. Doxorubicin was purchased from local representative of Pfizer Inc. (New York, USA).

*Cell culture.* Rat glioma cells of C6 line used in the experiments were obtained from the Collection of the Institute of Molecular Biology and Genetics, National Academy of Science of Ukraine (Kyiv, Ukraine). Cells were cultured in Dulbecco's modiﬁed Eagle's medium (DMEM, Sigma, St. Louis, USA) supplemented with 10% fetal bovine serum (Sigma). Cells were grown in CO_2_-incubator at 37°C, 5% CO_2_ and 95% humidity. The reseeding of cells was performed at a ratio of 1:5 once in 2-3 days.

*Determination of cytotoxic action of studied substances.* Cells were plated in 96- or 24-well plates (Greiner Bio-One GmbH, Kremsmünster, Austria). The substances under study were added at various concentrations immediately after cell seeding without the adaptation period. Counting of the cell number was carried out at regular intervals in the hemocytometer (counting chamber) using Trypan Blue dye (DV-T10282, Invitrogen, Life Technologies Corporation, Waltham, MA, USA) at 0.01% final concentration, 2 min after its addition to cell suspension. The dead cells uptake this dye due to their plasma membrane damage.

*Cell viability and proliferation assay*. MTT assay was used for assessing cell metabolic activity. NAD(P)H-dependent cellular oxidoreductases are reducing 3-(4,5-dimethylthiazol-2-yl)-2,5-diphenyltetrazolium bromide (MTT) to its insoluble formazan dissolved in the DMSO and measured by the colorimetric analysis as a purple color. After drug treatment, the MTT assay of viable cells was conducted in accordance with the manufacturer’s recommendations (Sigma). Product of the reaction was quantitatively determined by an Absorbance Reader BioTek ELx800 (BioTek Instruments, Inc., Winooski, VT, USA) at 620 nm wavelength.

*Light and fluorescent microscopy.* The living, apoptotic and necrotic cells were viewed under the inverted light microscope Biolam (LOMO, St Petersburg, Russian Federation). Images were processed with a fluorescent Zeiss microscope (Carl Zeiss, Jena, Germany) using AxioImager A1 camera, at ~ 400 times magnification in the relevant sections of the excitation and emission.

Rat glioma C6 cells were seeded on glass microscopic slides in the 24-well plates (Greiner bio-one). The substances were added in different concentrations in 24 hours after cell seeding. Material of cell nucleus was stained with the DNA-specific fluorescent dye Hoechst 33342 (Sigma). The cells were also stained by the poly-specific dye Acridine orange (AO, Sigma) (14). These fluorochromes were added to cultured cells at following final concentrations: AO – 0.3-1.0 µg/mL, Hoechst 33342-0.2-0.5 µg/mL, and the cells were incubated for 20-30 min.

*Flow cytometry*. For cell cycling and apoptosis study, C6 glioma cells treated with various agents were washed with phosphate buffered saline (PBS, pH 7.4), pelleted by centrifugation at 1000 rpm for 5 min at 4°C, and re-suspended in cold PBS (2 mln cells per 1 mL of the PBS). Then, cells were fixed via dropwise adding of cooled (-20°C) absolute ethanol (total volume 4 mL) with gentle mixing. The obtained fixed samples were kept at -20°C until use (no longer than 1 week). For conducting FACS analysis, cell samples were centrifuged at 1000 rpm for 5 min at 4°C, the supernatant was discarded, and the cell pellet was re-suspended in 1 mL of PBS. 100 uL of DNAase-free RNAase (Sigma) were added to that suspension, and samples were incubated at 37°C for 30 min. Then, each sample was supplemented with 100 uL of the Propidium iodide (Sigma, 1 mg/mL) and incubated at room temperature for 10 min. The samples were transferred to plastic Falcon tubes, and cell suspension was monitored at the FACScalibur ﬂow cytometer (BD Biosciences, Mountain View, CA, USA) and Summit v3.1 software (Cytomation, Inc., Fort Collins, CO, USA) was used for measuring the parameters of cell cycle and apoptosis.

Measurement of the Annexin V-positive (apoptotic) cells was performed by FACS analysis. Glioma C6 cells were treated as indicated in the ﬁgures. At the end of the experiment, the cells were detached from the bottom of the dish with trypsin-EDTA solution, washed twice with the PBS, and stained with Annexin V-FITC using apoptosis detection kit (BD Pharmingen, San Diego, CA, USA), according to the manufacturer's instructions. In particular, washed cells were incubated for 15 min in the Annexin V binding buffer containing 1/50 volume of FITC-conjugated Annexin V solution. Then, the samples were diluted twice with an appropriate volume of the Annexin V binding buffer and immediately measured on FL1/FL2 (FITC-PI) channel of the flow cytometer device (Becton Dickinson, Franklin Lakes, NJ, USA). The Annexin V-single positive cells were classified as apoptotic.

*Animal study.* All experiments with experimental rats were conducted under the control of the BioEthics Commission at Danylo Halytsky Lviv National Medical University (Protocol N2 dated by 16.02.2015) (15,16). Mature white laboratory rats with body mass of 200-220 g were kept on a standard fodder in animal facility with adequate lighting and temperature conditions.

Drugs were administered to animals every day in the morning before the first meal. Rats had an access to water all the time (24 h). Such a mode of administration of drugs is common in preclinical study of medicines (17). The experiment lasted for 10 days for animals that received doxorubicin and for 20 days for animals that received the synthetic compounds with anticancer potential. Doxorubicin was injected starting from the dose of 5.5 mg/kg, the compounds ID 3882 and ID 3833 – starting from the dose of 10.7 mg/kg, and the compound ID 3288 – starting from the dose of 24.3 mg/kg. The dose was gradually elevated by 1.5 times per 4 days in order to achieve a cumulative effect. The starting dose was equaled by 10% of maximum injected dose in experiments for LC_50_ determination (8). Experimental groups used in the study: 1 – control (intact animals, n = 20); 2 – doxorubicin injection (n = 20); 3 – ID 3288 injection (n = 20); 4 – ID 3882 injection (n = 20); 5 – ID 3833 injection (n = 20). The rats were euthanized on the 10th or 20th day by decapitation under thiopental anesthesia (17). Blood was used to obtain serum.

*Measurement of ROS and MDA in blood serum of treated rats*. The content of generated superoxide radical was measured by the oxidation of the cytochrome *c* (Sigma) defined as an optical density at 550 nm (18). The content of hydroxyl radical was measured in the incubation medium with 2-deoxy-D-ribose (Sigma) defined as an elevation of the malonic dialdehyde (MDA) by absorption at 532 nm (19). Means of both indicators were presented as the arbitrary units of changed extinction per 1 min in 1 mL of blood serum. The content of H_2_O_2_ was measured, as previously described (20). For measuring the malonic dialdehyde, 0.5 mL of 1% solution of the tiobarbituric acid in 50 mM NaOH and 0.5 mL of 2.8% solution of trichloracetic acid were added to the aliquots of blood serum. The mixture was kept for 20 min in boiling water bath cooled to room temperature, and optical density was determined at 532 nm (18).

Measurement of the enzymatic activity of the antioxidant system was conducted as follows. The activity of gluthatione peroxidase (GPO, EC 1.11.1.9) was measured spectrophotometrically at λ = 262 nm, as a quantity of the reduced gluthatione. It was expressed in the micromoles of reduced gluthatione per 1 min in 1 mL. Catalase activity (CAT, EC 1.11.1.6) was determined by measuring the decrease in the hydrogen peroxide concentration at 410 nm. The assay medium consisted of 1 mL Tris-HCl buffer solution (0.05 mmol, pH 7.8), 0.1 mL of blood serum sample, and 2 mL of 0.03% H_2_O_2_. The reaction was stopped after 10 min incubation by adding 1 mL of 4% ammonium molybdate. Spectrophotometric measurement was conducted at 410 nm, and the activity of blood serum CAT was expressed as the micromoles of H_2_O_2_/min per mg of protein. The activity of superoxide dismutase (SOD, EC1.15.1.1) was determined in the reaction of reduction of the nitrotetrazolium blue to the nitroformazan. The assay medium contained 0.1 mL of blood serum (1:10 dilution), 0.9 mL of distilled water, 0.5 mL of absolute ethanol, 0.25 mL of chloroform, and 0.3 g KH_2_PO_4_. The mixture was shaken vigorously, centrifugated (5000 rpm, 30 min), and 0.1 mL of NADPH and 0.05 mL of the incubation solution (37 mg EDTA, 330 mg p-iodonitrotetrazolium violet, 55 mg phenazine methosulfate were added to a supernatant and incubated for 10 min at 20°C. The absorbance was immediately read at 540 nm in a Stat fax. SOD activity was expressed in μmol/min per mg of protein. Н_2_S content was determined in the reaction with N,N-dimethyl-para-phenyldiamine in the presence of FeCl_3_ (21).

*Data analysis and statistics.* All experiments were repeated three times with three parallels in each variant. The analysis of variance (ANOVA) was used as a statistical test for comparison of groups. All data are presented as a mean ± SD. Results were analyzed using GraphPad Prism software. Statistical analyses were performed using *t* test or two-way analysis of variance (ANOVA). To examine differences between drug treatment responses, Bonferroni post-hoc tests were conducted. *P* values below 0.05 were considered as statistically significant.

## Results

### Cell viability assay and Trypan blue exclusion testing of cytotoxic action of 4-thiazolidinone derivatives

**Figure 2:**
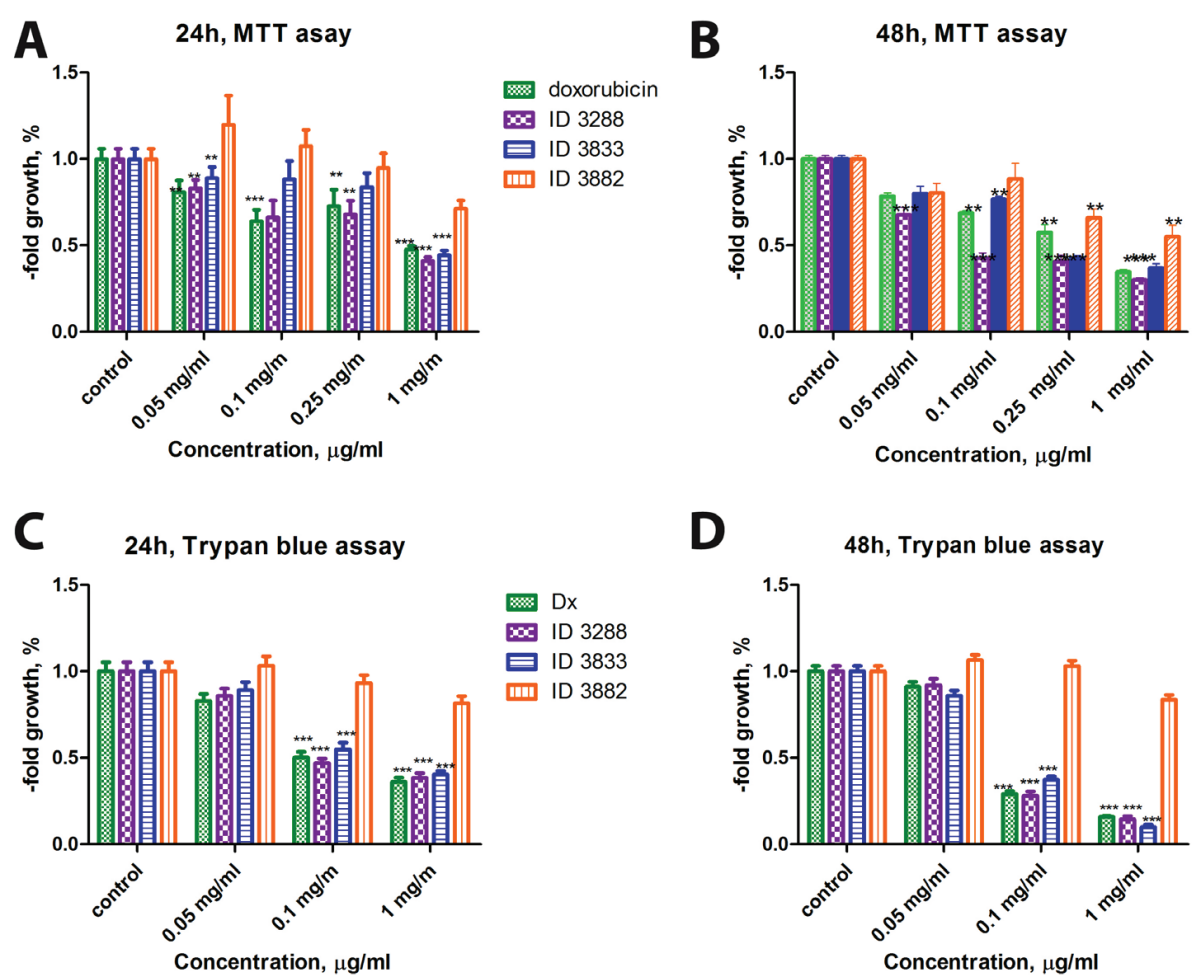
Dynamics in the number of alive rat glioma C6 cells under treatment for 24 h and 48 h with 4-thiazolidinone derivatives – compounds ID 3288, ID 3833, and ID 3882 compared with the action of doxorubicin (positive control). Negative control – 100% (untreated cells cultured for 24 h and 48 h, respectively). (**A**) – MTT assay 24h; (**B**) – MTT assay 48 h; (**C**) – Trypan blue assay 24 h; (**D**) – Trypan blue assay 48h. * – *P* ≤ 0.05; ** – *P* ≤ 0.01, *** – *P* ≤ 0.001 (difference compared with the negative control).

The MTT assay was applied for measuring the cytotoxic effect of the studied compounds ID 3288, ID 3882, ID 3833 and doxorubicin on rat glioma C6 cells. The compound ID 3288 was the most effective, and its activity was even higher (48 h treatment) than the activity of doxorubicin (Figure 2). The compound ID 3882 demonstrated some cytotoxicity only at a high dose (1 µg/mL) and at longer (48 h) treatment, while the cytotoxicity value of ID 3833 was between the values for ID 3288 and ID 3882.

In general, the pattern of the cytotoxic action of the studied compounds and doxorubicin obtained by Trypan blue exclusion test was similar to the pattern obtained by the MTT assay. Particularly, the compounds ID 3288 and ID 3833 demonstrated similar cytotoxicity as doxorubicin at both time points of measurement – 24 and 48 h (Figure 2). It should be noted that the compound ID 3882 did not change significantly the number of Trypan-blue positive (dead) cells even when used at a high dose (1 µg/mL).

These results correspond with the values of 50% inhibition concentration (IC_50_) determined by the MTT assay and 50% lethal concentration (LC50) determined by the Trypan blue exclusion test in rat glioma C6 cells treated for 24 and 48 h with the compounds ID 3288, ID 3833, ID 3882, and doxorubicin (positive control). The studied compounds showed a time dependent action, and the compound ID 3288 was the most cytotoxic, while the compound ID 3882 was non-toxic (Table 1).

**Table 1:** Values of IC_50_ determined by the MTT assay and of LC50 determined by the Trypan blue exclusion test in rat glioma C6 cells treated for 24 and 48 h with the compounds ID 3288, ID 3833, ID 3882, and doxorubicin (positive control).

Drugs	MTT (IC_50_, μg/mL)		Trypan blue (LC50, μg/mL)	
**24 h**	**48 h**	**24 h**	**48 h**
**Dox**	0.71	0.51	0.84	0.07
**3288**	0.43	0.10	0.13	0.07
**3833**	0.84	0.23	0.89	0.08
**3882**	non-toxic	0.98	non-toxic	non-toxic

### Cytomorphological characteristics of glioma C6 cells treated with 4-thiazolidinone derivatives

**Figure 3:**
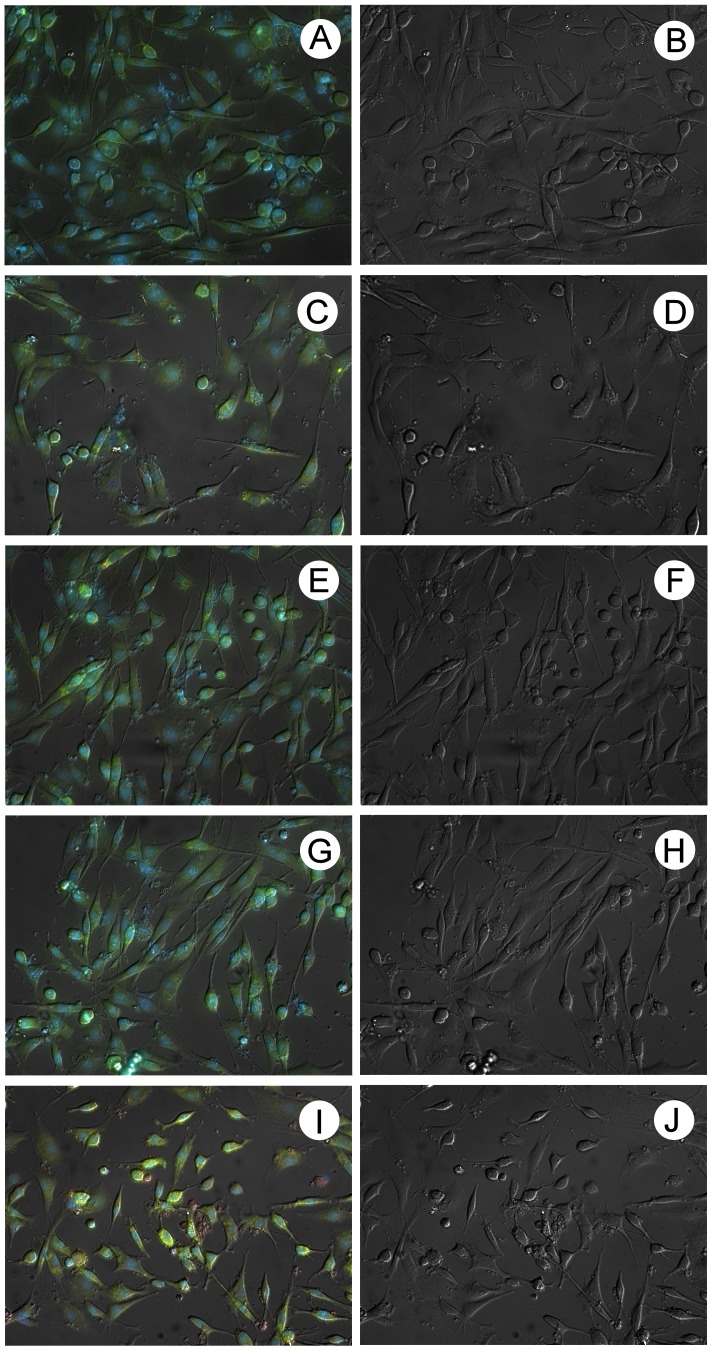
Rat glioma C6 cells after 24 h treatment with the studied compounds (**A**), (**B**) – control; (**C**), (**D**) – ID 3288; (**E**), (**F**) – ID 3833, (**G**), (**H**) – ID 3882) and doxorubicin (positive control, (**I**), (**J**), all used in 1 µg/mL dose. Left – fluorescent image of treated cells (blue color – staining with fluorescent DNA-specific dye Hoechst-33342, red and green color – staining with poly-specific fluorescent dye Acridine orange). Right – DIC image of treated cells.

The microscopic study of the cytomorphological characteristics of the treated glioma C6 cells demonstrated that doxorubicin and compound ID 3288 used at 1 µg/mL dose caused the most drastic changes in cell intactness, namely a decrease in cell density *in vitro,* a decrease in cell size, probably due to apoptotic condensation of cell body, and appearance of cell debris (Figure 3). Besides, doxorubicin induced a development of red color staining of cells in the presence of the Acridine orange dye (Figure 3). Other compounds (ID 3288 and ID 3833) impaired cell morphology to a lesser extent than the ID 3288.

### FACS analysis of changes in cell cycling induced by 4-thiazolidinone derivatives

FACS analysis of cell cycling and apoptosis is a sensitive test for evaluating the effect of various agents on cell functioning and viability, and we used this approach to measure the cytotoxic action of the studied 4-thiazolidinone derivatives. Its results are presented in Table 2. There was a clear time and dose dependence of cytotoxicity (number of pre-G1 apoptotic cells) of these agents (excluding the compound ID 3882) targeting rat glioma C6 cells. The ID 3882 did not disturb cell cycling considerably, and this is in agreement with the results of MTT assay, Trypan blue exclusion test, and cytomorphological study.

**Table 2:** Results of typical FACS analysis of the number of cell cycle phases – pre-G1 (apoptotic), G1, S, and G2/M – in rat glioma C6 cells treated with compounds ID 3288, ID 3882, ID 3833 and doxorubicin (positive control) used in 0.1 and 1.0 µg/mL doses for 24 and 48 h

**24 h**	**Control**	**Doxorubicin**				**3288**			**3833**			**3882**		
		0.1 µg/mL		1.0 µg/mL		0.1 µg/mL	1.0 µg/mL		0.1 µg/mL	1.0 µg/mL		0.1 µg/mL		1.0 µg/mL
**Pre-G1**	3.83	4.55		15.89		3.93	53.75		3.79	26.51		3.12		4.39
**G1**	63.4	58.96		56.37		62.04	31.16		60.62	50.49		60.81		64.98
**S**	11.54	5.07		13.51		11.5	4.56		12.09	6.96		12.06		8.41
**G2**	20.13	30.67		15.16		20.95	10.86		21.46	16.13		21.53		20.44
**48 h**	**control**		**DMSO**		**Doxorubicin**			**3288**			**3833**		**3882**	
					0.1 µg/mL			0.1 µg/mL			0.1 µg/mL		0.1 µg/mL	
**Pre-G1**	3.89		0.81		18.98			87.84			5.41		7.71	
**G1**	62.33		72.92		52.39			10.12			59.34		66.01	
**S**	13.97		7.99		6.13			0.84			11.14		9.39	
**G2**	19.96		16.71		22.41			1.28			20.51		16.85	

### FACS analysis of apoptosis induced by 4-thiazolidinone derivatives

**Figure 4:**
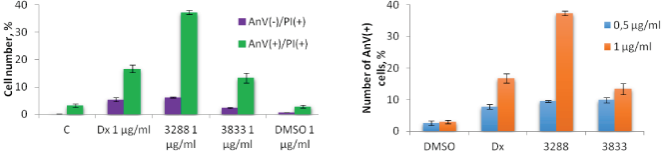
Effect of compounds ID 3288, ID 3833 and doxorubicin in 0.5 and 1.0 µg/mL concentrations (24 h) on induction of apoptosis in rat glioma C6 cells. Results of flow cytometry of cells after Annexin V (AnV) and Propidium iodide (PI) double staining.

The results presented above on the amount of the pre-G1 phase (Table 2) correspond with the results of measuring the Annexin V-single positive cells classified as apoptotic ones (Figure 4).

Flow cytometry study conducted after double staining (Annexin V+/Propidium Iodide+) of rat glioma C6 cells treated for 24 h with the compounds ID 3288, ID 3833, and doxorubicin (positive control) clearly demonstrated that the compound ID 3288 dose-dependently (0.5 and 1.0 µg/mL) induced apoptosis in these cells (Figure 4). The number of pre-G1 cells treated with ID 3288 (Table 2) was comparable with the sum of numbers of apoptotic (AnV+/PI+) and necrotic (AnV-/PI+) cells (Figure 4). An increased number of AnV+/PI+ cells under treatment with ID 3288 was bigger than in the case of doxorubicin treatment, and the effect of ID 3833 was weaker than the effect of doxorubicin, both used at 1.0 µg/mL concentration. DMSO (used for dissolving the 4-thiazolidinone derivatives under study) in both doses (0.5 and 1.0 µg/mL) was relatively non-toxic for glioma cells (Table 2), and it did not significantly affect cell cycling or induce apoptosis (Figure 4). These results suggest that the compound ID 3288 can potentially be used for treatment of mammalian gliomas.

### Measurement of biochemical mediators of the action of 4-thiazolidinone derivatives in the serum of rats

**Figure 5:**
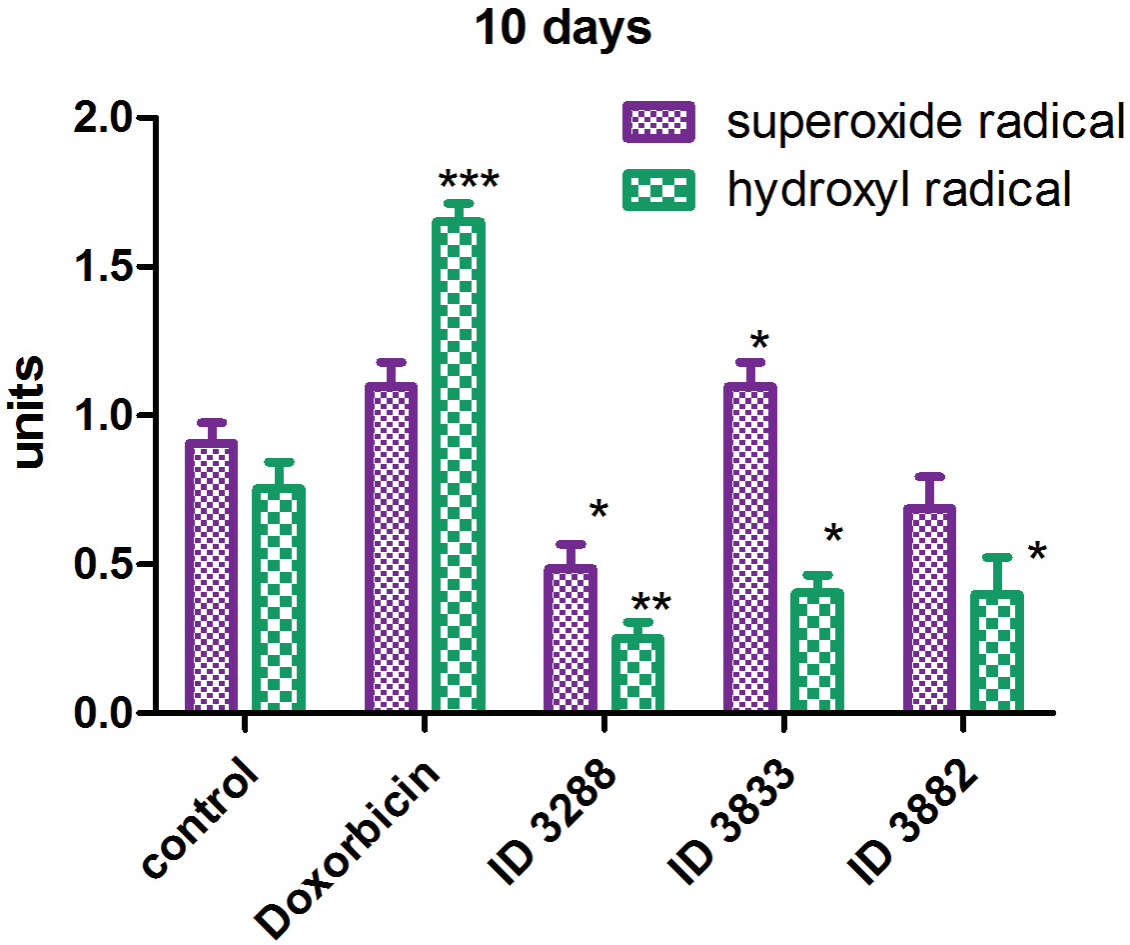
Concentration of superoxide radical and hydroxyl radical in the serum of rats injected with doxorubicin and the compounds ID 3288, ID 3882, and ID 3833. * – *P* ≤ 0.05; ** – *P* ≤ 0.01, *** – *P* ≤ 0.001 (difference compared with the control).

We also measured the level of ROS in the serum of rats. The level of superoxide radical in serum was not changed by doxorubicin, while it was decreased by 46% and 24% after the injection of the compounds ID 3288 and ID 3882, respectively (Figure 5). The compound ID 3833 did not change the level of superoxide radical significantly.

An injection of the doxorubicin led to a significant (2.2 times) increase in the content of the hydroxyl radical in the serum of treated rats, while the injection of all studied 4-thiazolidinone derivatives (ID 3288, ID 3882, ID 3833) decreased its content by 2-3 times (Figure 5).

**Figure 6:**
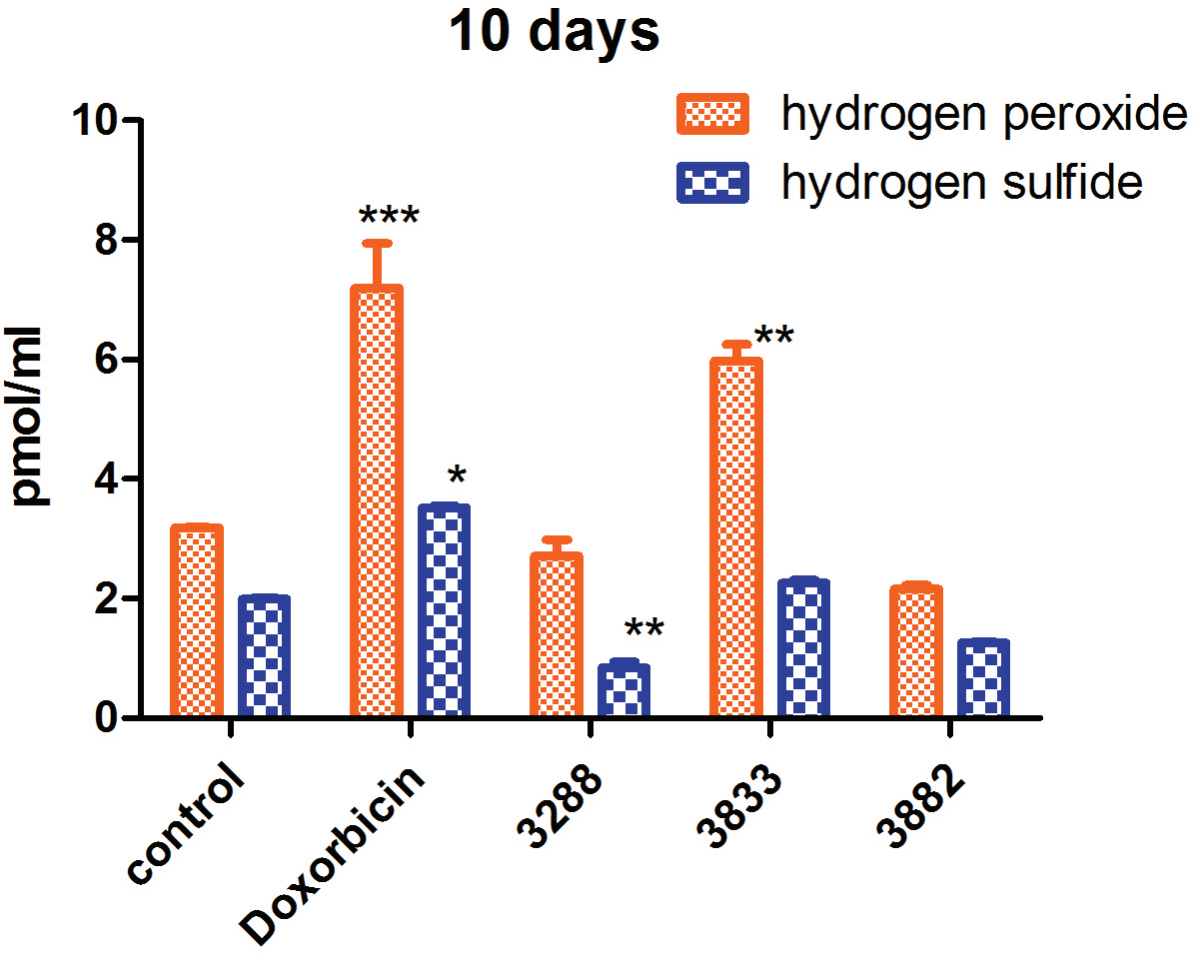
Concentration of hydrogen peroxide and hydrogen sulfide in the serum of rats injected with doxorubicin and the compounds ID 3288, ID 3882 and ID 3833. * – *P* ≤ 0.05; ** – *P* ≤ 0.01, *** – *P* ≤ 0.001 (difference compared with the control).

In general, the ability of the compounds ID 3288, ID 3882, ID 3833 and doxorubicin to induce the production of hydrogen peroxide is similar to their ability to induce superoxide and hydroxyl radical (Figure 6). While doxorubicin and the ID 3833 increased approximately twice the content of the hydrogen peroxide in the serum of treated rats, the ID 3288 did not change it significantly, and ID 3882 decreased such content by 30%.

Here, we found that doxorubicin increased the content of H_2_S by 75%, while the compounds ID 3288 and ID 3882 decreased such content by 57% and 35%, respectively, and the compound ID 3833 did not affect it significantly (Figure 6).

**Figure 7:**
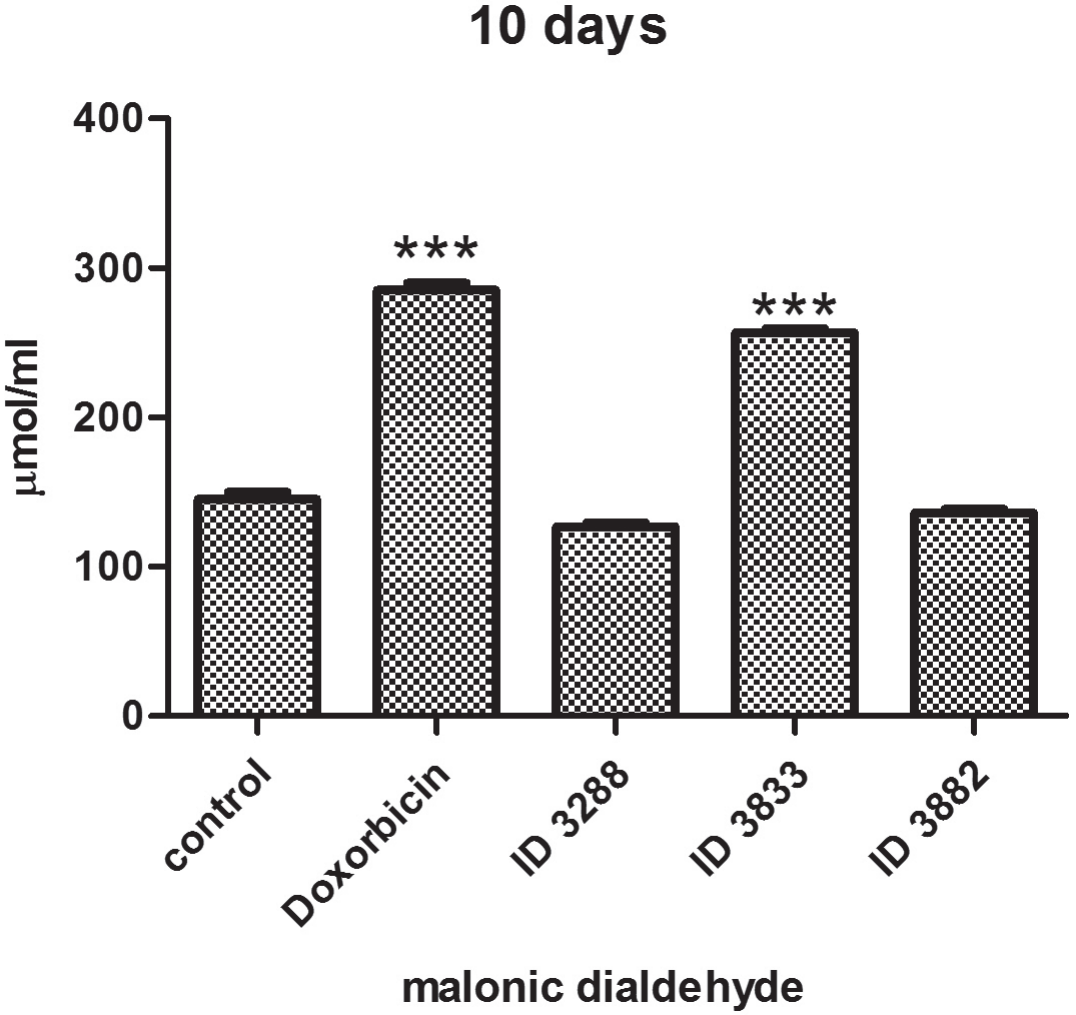
Concentration of the malonic dialdehyde in the serum of rats injected with doxorubicin and the compounds ID 3288, ID 3882, and ID 3833. *** – *P* ≤ 0.001 (difference compared with the control).

While doxorubicin and the compound ID 3833 increased twice the content of the malonic dialdehyde in the serum of treated rats, the ID 3288 and ID 3882 did not affect that content (Figure 7).

### Measurement of the activity of enzymes of the antioxidant system in the serum of rats

**Figure 8:**
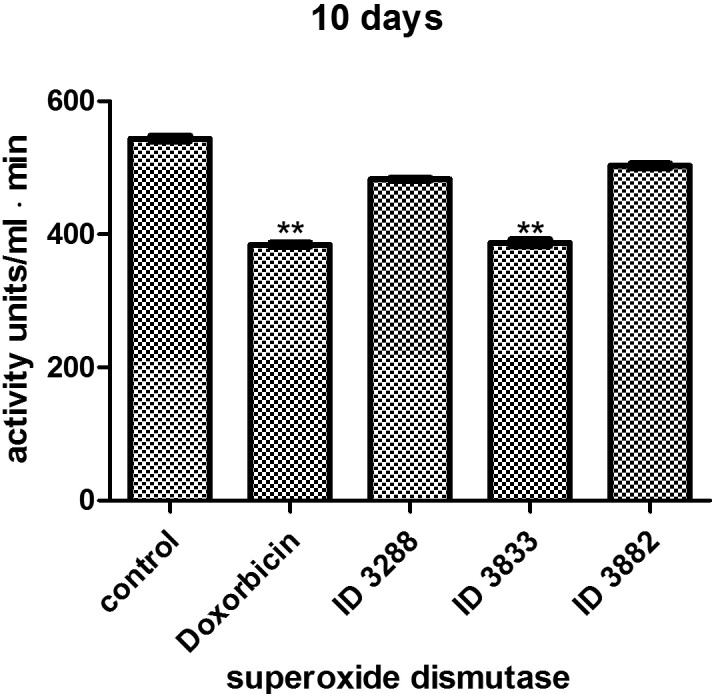
Superoxide dismutase activity in the serum of rats injected with doxorubicin (Dox) and the compounds ID 3288, ID 3882, and ID 3833. * – *P* ≤ 0.05; ** – *P* ≤ 0.01, *** – *P* ≤ 0.001 (difference compared with the control).

**Figure 9:**
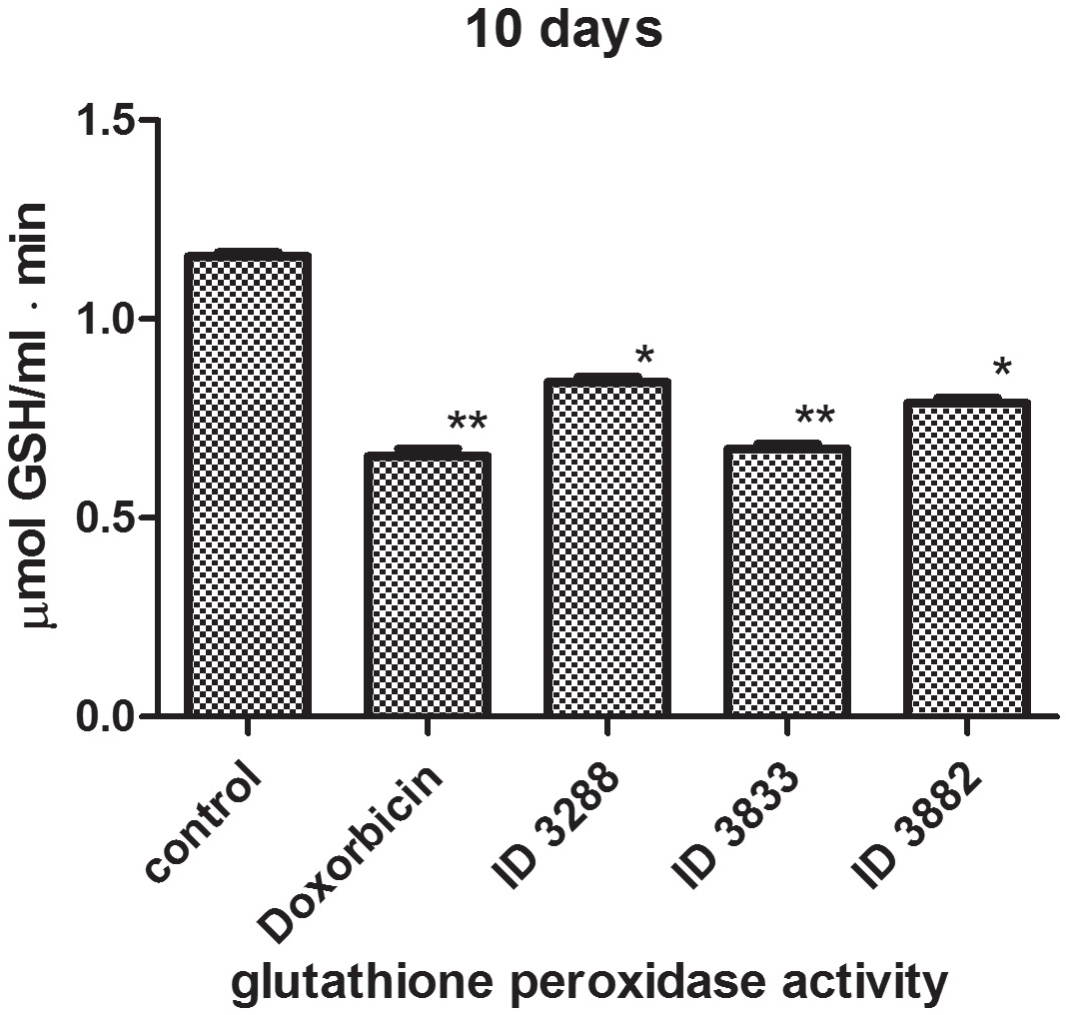
Glutathione peroxidase activity in the serum of rats injected with doxorubicin (Dox) and the compounds ID 3288, ID 3882, and ID 3833. * – *P* ≤ 0.05; ** – *P* ≤ 0.01, *** – *P* ≤ 0.001 (difference compared with the control).

**Figure 10:**
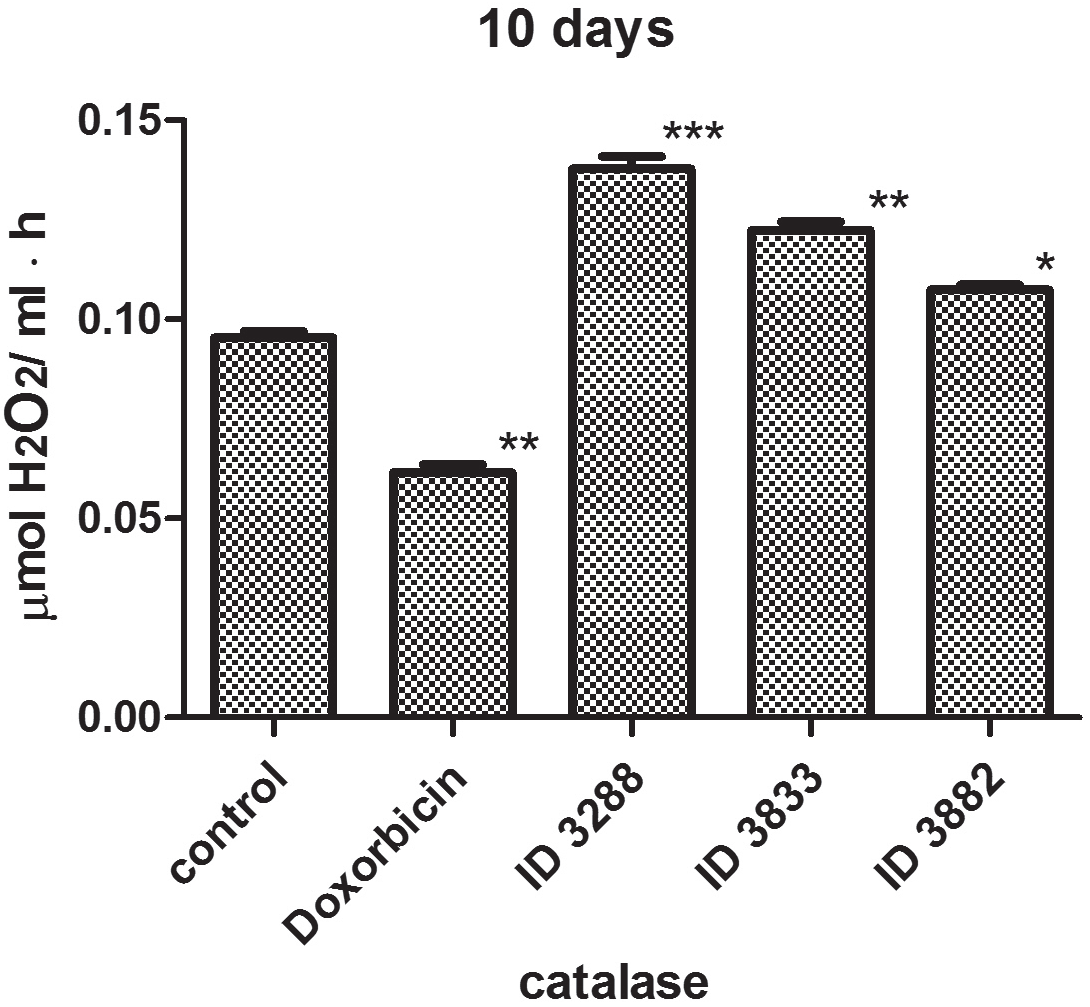
Catalase activity in the serum of rats injected with doxorubicin (Dox) and the compounds ID 3288, ID 3882, and ID 3833. * – *P* ≤ 0.05; ** – *P* ≤ 0.01, *** – *P* ≤ 0.001 (difference compared with the control).

The activity of enzymes of the antioxidant system – SOD, Cat and GPO – was measured in the serum of treated rats. The activity of the GPO was decreased under the action of all studied anticancer compounds, while the activity of SOD was decreased only by doxorubicin and the ID 3833 (Figure 8,9). In general, these data correspond with the results obtained when the ROS were measured in the same variants of the experiment. The dynamics of the Cat activity under the action of doxorubicin (35% decrease) was similar to the dynamics detected for the SOD and GPO activities. However, the effect of the ID 3288 and ID 3833 was different since they increased the activity of Cat by 40% and 20%, respectively (Figure 8,10).

Thus, the action of the compounds ID 3288, ID 3833 and ID 3882 was accompanied by a lower increase in ROS content and a lower decrease in the activity of enzymes of the antioxidant system, compared with such actions of the doxorubicin.

## Discussion

We used four different approaches for evaluating toxic action of the 4-thiazolidinone derivatives (compounds ID 3882, ID 3833, ID 3288) and doxorubicin on rat glioma C6 cells: 1) MTT (cell viability and proliferation) assay; 2) Trypan blue exclusion test for cytotoxicity; 3) cytomorphological (microscopic) study of cell intactness; 4) study of cell cycling and apoptosis (flow cytometry). A specific ranking of cytotoxicity of the applied drugs was formulated taking into account the results of these tests.

Evaluation of the vitality and survival (MTT assay) of treated cells showed the following ranking of increasing toxicity of 4-thiazolidinone derivatives toward the glioma cells: ID 3882<ID 3833<ID 3288 ≈ doxorubicin. This ranking was found to be true when the MTT assay was applied for monitoring the results of 24 h treatment with median (0.1-0.25 µg/mL) doses of the compared agents, while at 48 h treatment with the same doses, it was somewhat changed: ID 3882<ID 3833<doxorubicin<ID 3288. There was no big difference between the action of the studied drugs used in low (0.05 µg/mL) and high (1.0 µg/mL) doses, with an exception of the compound ID 3882 which demonstrated very low toxicity toward the glioma cells.

The ranking of the toxic action (Trypan blue exclusion test) of 4-thiazolidinone derivatives and doxorubicin on rat glioma C6 cells was the following: ID 3882<ID 3833 ≈ ID 3288 ≈ doxorubicin at both 24 and 48 h duration of cell treatment. Thus, it was similar to that observed when the MTT assay for cell vitality and survival was used (see above).

4-thiazolidinone derivatives used in this study were also tested for their toxicity toward other mammalian tumor cells, such as murine leukemia L1210 cells, human T leukemia Jurkat cell line (6), and human melanoma WM793 cells (our paper under preparation). The compound ID 3833 was found to be the most cytotoxic (6). As shown in this study, the ID 3288 was the most cytotoxic for rat glioma cells, compared with the ID 3833 and 3882. Thus, one can suggest an existence of specific action of the ID 3288 on rat glioma cells. It was also shown that the ID 3833 was as toxic as doxorubicin for human embryonal kidney HEK-293 cells, while the ID 3288 and 3882 were not toxic for these cells (our unpublished data).

It was rather complicated to quantify the cytotoxicity levels by using the cytomorphological (microscopic) studies. However, the results of these studies, as well as that of previous tests, showed the highest toxicity of the compound ID 3288 and the lowest toxicity of the ID 3882 for rat glioma C6 cells.

The same conclusion can be done based on the results of FACS analysis on the ratio of the apoptotic (pre-G1) glioma C6 cells appearing under their treatment with studied agents. While at 24-h cell treatment with this compound in 1.0 µg/mL dose, the pre-G1 peak on the cytofluorogram reached 53.75%, a longer duration (48 h) treatment with 10 times lower dose (0.1 µg/mL) of the ID 3288 led to massive (87.84%) death of the glioma cells, suggesting their secondary necrosis. This suggestion corresponds with the results of Trypan blue exclusion test measuring the necrotic cells. Thus, the glioma cells treated with the compound ID 3288 really die and are not simply metabolically suppressed. The results of the cytomorphological study of such cells also demonstrated that they had a decreased size and round form, and, moreover, they frequently possessed a damaged morphology (surface). Besides, signs of lysosome activation were observed in their cytosol regions as red staining by the Acridine orange (14). The action of the ID 3833 and ID 3288 was less destructive for the glioma cells. As a rule, changes in cell vitality and survival, as well as cell damage, are secondary to the deregulation of cell cycling and development of the apoptotic characteristics in cells (22).

Taking into account the results of our comparative studies of the cytotoxic action of three different 4-thiazolidinone derivatives (ID 3288, ID 3833 and 3882) and doxorubicin (positive control), one can ask a question – why the ID 3288 is the most damaging agent for rat glioma cells and the ID 3833 is the next in this ranking, while the ID 3882 is relatively non-toxic agent. It should be noted that in the structure of the ID 3288 and ID 3833, opposite to the ID 3882, Br atom is present in 5th position of the isatin fragment of their molecules, and the phenyl substituent is present in the 3rd position of the pyrazoline cycle of the ID 3288. However, additional investigation should be performed for more detailed analysis of structure-functional interrelations of the 4-thiazolidinone derivatives.

We found in the *in vivo* experiments that the compounds ID 3288 and ID 3833 induced the effects typical for the action of the doxorubicin. These are an increase in the level of the ROS and a decrease in the activity of the enzymes of the antioxidant system. It is evident that a proper modulation of the level of induced ROS and of the antioxidant activity might be a useful strategy to decrease the negative consequences of general toxicity accompanying the action of most anticancer drugs (9,12). This could be achieved by the combination of chemotherapy and antioxidants (10,13), although the application of such approach in cancer treatment has been criticized (23,24).

The main mechanism of the antioxidant defense is not based on a direct inhibition of reactions of free radical oxidation by various antioxidants. It mostly depends on a precise regulation of interrelations between pro- and antioxidant systems and a support of the antioxidant status in the organism via a flexible regulation of the metabolic processes (12,13,24-26). The negative side effects of anticancer drugs are related to a disturbance in the system of free radical processes, whose excess activation cannot be neutralized by a system of the antioxidant defense (9,11,25,26).

Our experiments show that doxorubicin induces an elevation of the superoxide and hydroxyl radicals, which are highly toxic and whose action is considered to be one of the main reasons of negative side effects of this antibiotic in the organism (9-12). Significantly lower contents of these radicals in the serum of rats treated with the compounds ID 3288 and ID 3882, compared with such contents found in the serum of rats treated with doxorubicin and compound ID 3833, are in agreement with our previous results of measuring the biochemical indicators (activity of creatine kinase, lactate dehydrogenase, aspartate aminotransferase, and alanine aminotransferase) of the cardiotoxicity in rats (2,3).

H_2_O_2_ was also strongly induced by doxorubicin and compound 3833, and not changed by the compounds 3288 and 3882. Besides, the level of malonic dialdehyde was increased by doxorubicin and the compound 3833, but not by the compounds 3288 and 3882. The malonic dialdehyde is considered to be an integrated indicator of ROS production under the action of various stressing agents, including anticancer drugs (18).

General pattern of changes of the H_2_S in the serum of rats treated with the compounds ID 3288, ID 3882, ID 3833, and doxorubicin was similar to the patterns of changes of specific ROS and malonic dialdehyde. That is an elevation of the content of H_2_S under the action of doxorubicin and a decrease of such content by the compounds ID 3288 and ID 3882. However, while the ID 3833 increased the level of such ROS as H_2_O_2_, superoxide radical and the malonic dialdehyde, this compound did not influence significantly the level of H_2_S. After first description of H_2_S role as a regulator of a variety of such processes, it has been intensively studied (21). H_2_S enhanced the apoptotic death of the mammalian cells *in vitro* using the mitochondrial pathway for the activation of caspase 3 and specific MAP-kinases (21). The pro-apoptotic action of H_2_S in high millimolar doses is accompanied by the generation of ROS and a decrease in the gluthatione concentration (21). Thus, the action of doxorubicin and the ID 3833 on H_2_S level correlates with a potential proapoptotic effect of this mediator.

The determined changes in the enzymatic system of the antioxidant defense in rat glioma C6 cells treated with 4-thiazolidinone derivatives were the following: activity of SOD was decreased under the action of the doxorubicin and the compound ID 3833, but not by other compounds – ID 3288 and ID 3882, and the GPO activity was reduced at the action of all used compounds and doxorubicin. While only doxorubicin caused a reduction of the Cat activity, and the 4-tiazolidinone derivatives did not affect it.

Most metabolites and signaling mediators that appear in tissues and organs can also be found in the blood stream. Since the oxidants measured in our study are generated by cells, an increase in their content is also expected in blood. At present, we do not have direct experimental data on the penetration of the blood brain barrier by the compounds under study. Recently, it has been shown that similar heterocyclic compounds (1,3,4-Thiadiazolines) crossed the blood-brain barrier (BBB) and they were considered as anticancer drugs in the preclinical xenograft models (27).

Thus, we demonstrated that novel 4-thiazolidinone derivatives – the ID 3288, ID 3833 and ID 3882 – differ significantly in their cytotoxic action *in vitro* on rat glioma C6 cells. The ID 3288 possessed the highest cytotoxicity that was comparable with such action of the doxorubicin, while the ID 3882 was relatively non-toxic for these cells. *In vivo* action of these compounds and doxorubicin (positive control) on the level of ROS (potential mediators of toxic effects) in the blood of rats revealed the most prominent elevation of their level at the doxorubicin action, while the ID 3288 and ID 3882 were not increasing ROS level and the ID 3833 was in between the effects of the doxorubicin and these two 4-thiazolidinone derivatives. The last agents also decreased the activity of enzymes of the antioxidant system (SOD, GPO, and Cat) to much lower extent, compared with the action of doxorubicin.

These data could be of interest for considering a combined application of 4-thiazolidinone derivatives and antioxidants for treatment of tumor-bearing animals, although presently we can nominate these derivatives only as putative anticancer drugs. The next steps in our studies will be focused at immobilization of the most effective 4-thiazolidinone derivatives (probably, the ID 3288) on the bio-functionalized nanoparticles in order to enhance drug delivery and antineoplastic action, as well as to improve their capability to circumvent blood-brain barrier in tumor-bearing animals (28).
